# Impacts of climate change and human activities on the water discharge and sediment load of the Pearl River, southern China

**DOI:** 10.1038/s41598-020-73939-8

**Published:** 2020-10-07

**Authors:** Xing Wei, Shuqun Cai, Peitong Ni, Weikang Zhan

**Affiliations:** 1 State Key Laboratory of Tropical Oceanography, South China Sea Institute of Oceanology, Chinese Academy of Science, Guangzhou, 510301 China; 2 Southern Marine Science and Engineering Guangdong Laboratory (Guangzhou), Guangzhou, 511458 China; 3 Guangdong Key Lab of Ocean Remote Sensing, Guangzhou, 510301 China; 4 Innovation Academy of South China Sea Ecology and Environmental Engineering, Chinese Academy of Sciences, Guangzhou, 510301 China; 5 Guangdong Research Institute of Water Resources and Hydropower, Guangzhou, 510610 China

**Keywords:** Climate sciences, Environmental sciences, Hydrology, Ocean sciences

## Abstract

Global climate change and human activities have important effects on the water discharge and sediment load of the Pearl River. In this study, the water discharge and sediment load were investigated by using hydro-meteorological data from 1954 to 2018. The linear regression, Mann–Kendall abrupt test and double mass curve were employed to detect trends and abrupt change-points in water discharge and sediment load and to quantify the effects of climate change and human activities on water discharge and sediment load. The results revealed that the annual sediment load exhibited a significant decreasing trend at a rate of − 2.24 × 10^4^ t/year, regardless of water discharge, and an abrupt change occurred in 1998. Human activities, especially dam construction contributed 96% to this change, while 4% was due to climate change. El Niño/Southern Oscillation (ENSO) events are often associated with low precipitation, resulting in low water discharge and sediment load, indicating that changes in ENSO periodicity could affect the inter-annual periodic variations of water discharge and sediment load. As population and economy boom, more dams are being built in the Pearl River basin, and special attention should be paid to the management and mitigation of the effects of dams on sediment load.

## Introduction

Water discharge (WD) and sediment load (SL) into the sea are dominant factors controlling beach processes, delta and estuarine evolution, and coastal zone ecological environments^1–3^. Therefore, understanding variations of WD and SL from the rivers to estuaries and oceans has been set as one of the goals of the International Geosphere–Biosphere Programme and its core project, Land Ocean Interaction in the Coastal Zone^4^. It has been concluded that climate change and human activities are the most important factors influencing riverine WD and SL^4–6^. Scientific, observations have indicated that the global surface temperature has increased by approximately 0.8 °C with a significant upward trend over the past 30 years and a rate greater than 0.2 °C per decade^7^. Global annual precipitation has also increased significantly at a rate of approximately 0.2 mm/year (P < 0.001)^8^. This global climate change has influenced global and regional hydrological cycles. It has estimated that global runoff could increase by 4% based on an increase in global temperature of 1 °C^9^. Additionally, human activities, such as land use changes, freshwater extraction, and dam construction, have intensified over past several decades, often resulting in significant influences on river systems^4,6^. Decreased sediment loads have caused erosion in many river deltas, including the Mississippi River in United States^10^, Mekong River in Vietnam^11^, Yellow River^12^ and Yangtze River^13^ in China. The response of water discharge and sediment load to climate change and human activities have become hot topics, garnering significant attention worldwide^14–17^. However, the causes for changes in WD and SL differ from river to river and vary over time. Therefore, it is necessary to expand and update knowledge regarding these influences for specific rivers, particularly large rivers, to aid in global and regional environmental management.

**Figure 1:**
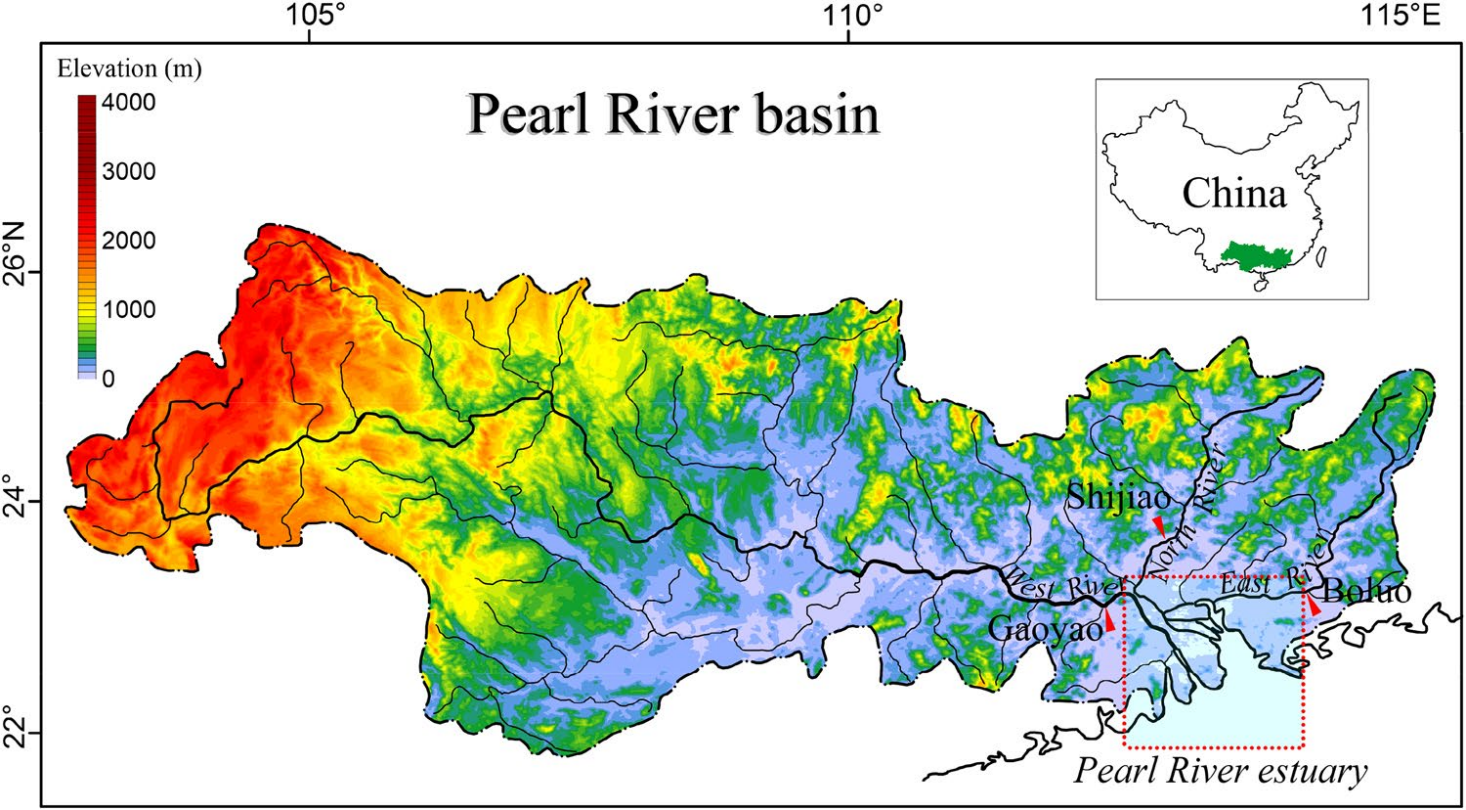
Index map of the Pearl River and is estuary (modified from Bulletins of Chinese River Sediment, https://www.mwr.gov.cn/sj/). The red triangle indicates the locations of hydrological stations. The color scale of the relief map shows the land elevation above the sea level in meter and the Digital Elevation Model (DEM) data are available at https://www.ngdc.noaa.gov/mgg/.

The Pearl River (PR) (Fig. 1) is the second largest river (after the Yangtze River) in China and the second largest river (after the Mekong River in Vietnam) that draining into the South China Sea in terms of annual WD. The Guangdong-Hong Kong-Macao Greater Bay Area, which is located in the Pearl River delta, is one of the most important economic centers in China. Base on the combined effects of climate change and human activities, the WD and SL of the PR have changed significantly over time. Many studies have been carried out to understand the variability of WD and SL of the PR^18–22^. Some studies have investigated the spatial and temporal variations of WD and SL in the Pearl River basin (PRB). Some attention has been paid to the impact of the soil conservation, deforestation, and dam construction on SL in the PRB. However, most of these studies have primarily focused on the influence of human activities or climate change impacts. More importantly, the quantification of climatic and anthropogenic effects on WD and SL in the PRB have received little attention. In the PR, similar to many other rivers, natural oscillations in the hydrological cycle and the processes influencing such oscillations must be distinguished before possible anthropogenic impacts can be analyzed accurately. The El Niño/Southern Oscillation (ENSO) is a result of ocean–atmosphere interactions on a macro spatial scale and is treated as the strongest inter-annual signal of climatic changes^23^. Thus, ENSO can be used as a specific parameter to characterize global climate change. Numerous studies have shown that ENSO-driven changes in temperature and precipitation correlate well with mean annual and seasonal river discharge^23–25^. Additionally, as global climate conditions changed, the frequency of global ENSO events increased during the late 1970s^26^. As a result, ENSO has become one of the most potential source of natural variability in the PRB. However, the effects of ENSO events on WD and SL in the PR have rarely been examined. Hence, this study, aimed to (1) provide updated estimates of WD and SL from the PR into the sea, (2) give an in-depth explanation of the influences of ENSO events on WD and SL, and (3) give a quantitative evaluation of the contributions of climate change and human activities to changes in WD and SL at the basin scale. We believe this study will provide a better understanding of natural and anthropogenic contributions to major river water and sediment transport processes, which will provide scientific guidelines for global river management.

## Result

### Impact of climatic changes

**Figure 2:**
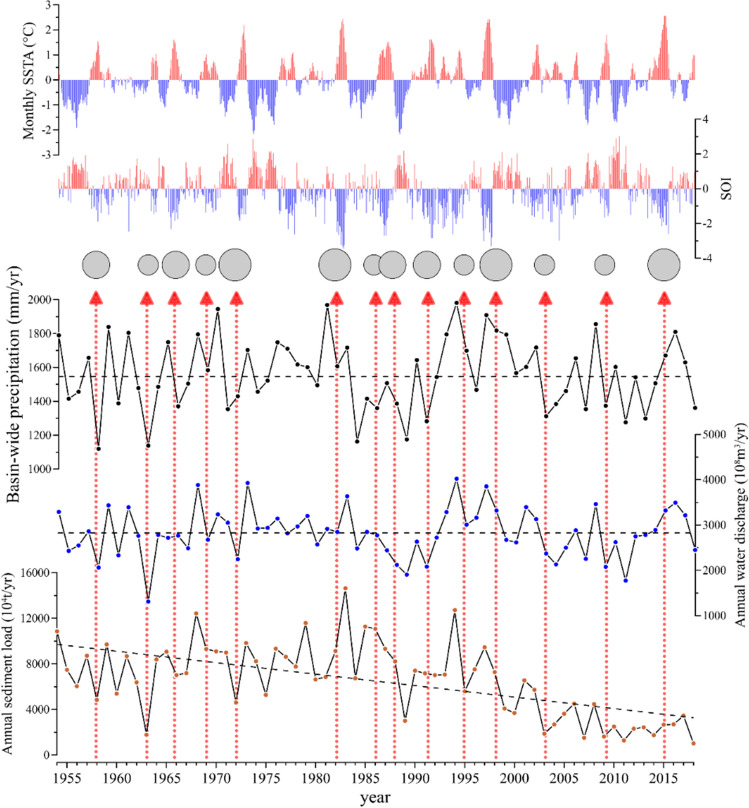
Time series of monthly SSTA, SOI, annual basin-wide precipitation (solid line) in the Pearl River basin, and water (solid line with dots) and sediment (dashed line) discharge into the sea over the past 65 years. Monthly sea surface temperature anomalies (SSTA) in the Niño 3.4 region and SOI values (1954–2009) were collected from https://www.ncdc.noaa.gov/teleconnections/. ENSO events were identified based on sustained positive SSTA and negative SOI values (shaded areas). The bubbles indicate years with ENSO events and their sizes correspond to the strength of ENSO events. The monthly and annual precipitation over the entire PR drainage basin and various source areas are given in the form of area-weighted average values, which were calculated from data recorded at 42 rain gauges spread across the river basin. The data were derived from the Chinese Meteorological Administration. The WD and SL data were collected from the three main gauging stations of the Pearl River System, namely Gaoyao station on the West River, Shijiao station on the North River, and Boluo station on the East River (Fig. 1). The data were derived from the Bulletins of Chinese River Sediment. The selected stations are located at tidal limits and the relationship between water levels and WD downstream is influenced by tides. Therefore, the WD and SL of these three gauging stations represent the discharges from the PRB into the sea.

ENSO events are closely linked to the patterns of flood and drought in different areas around the world^27^. They also strongly affect local- and regional-scale climates base on teleconnections affecting coupled ocean–atmosphere and land systems^23,24^. In the PRB, the average annual precipitation over the period of 1954 to 2018 was 1559 mm, and annual precipitation varied between 1120 and 1981 mm. The warm phases of monthly sea surface temperature anomalies (SSTA) were closely related to low precipitation in the PRB (Fig. 2). Large precipitation variations (> 5%) were commonly associated with ENSO years, such as in 1958, 1963, 1966, 1982, 1991, 1995, 2003, 2009, and 2015. This is mainly due to the fact that changes in sea surface temperature (SST) values stem from the equatorial Pacific Ocean trade winds, which feed moisture back into the atmosphere and eventually shift the pattern of regional precipitation in the PRB. Therefore, SSTA warm phases often result in low precipitation in the PRB. These results are consistent with those presented by previous studies^28,29^.

Since the late 1970s, global ENSO events have become stronger and more frequent^26^. Most of the low precipitation years in the PRB were closely associated with moderate and strong ENSO events (Fig. 2). Prior to1970s, the precipitation in the PRB had strong inter-annual variability (over 337 mm). However, inter-annual variations became milder as strong ENSO events occurred more frequently in subsequent years. The average annual precipitation in the river basin from 2000 to 2018 was only 1525 mm, which is approximately 11% less than that the 1990s. These correlations between ENSO events and lower regional precipitation indicate that regional precipitation is strongly affected by global climate systems.

**Figure 3:**
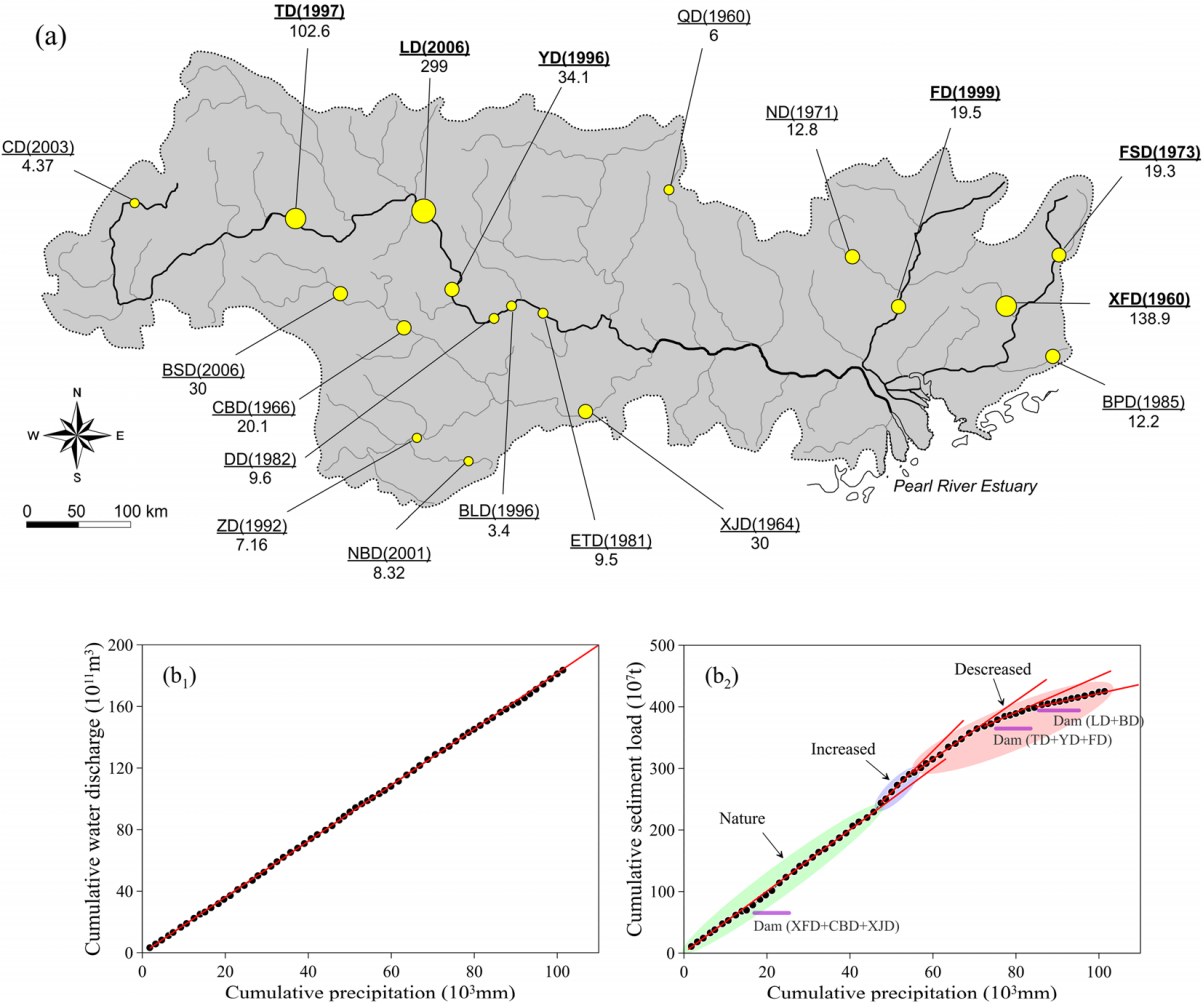
Main large dams in the Pearl River basin (modified from Ref.^20^) (**a**). Cumulative precipitation compared to water discharge and sediment load (**b**). Dam sites are denoted by circles with the name of the dam, year of dam closure in parentheses, and storage capacity of the corresponding reservoir (unit in × 10^9^ m^3^) under each name.

**Figure 4:**
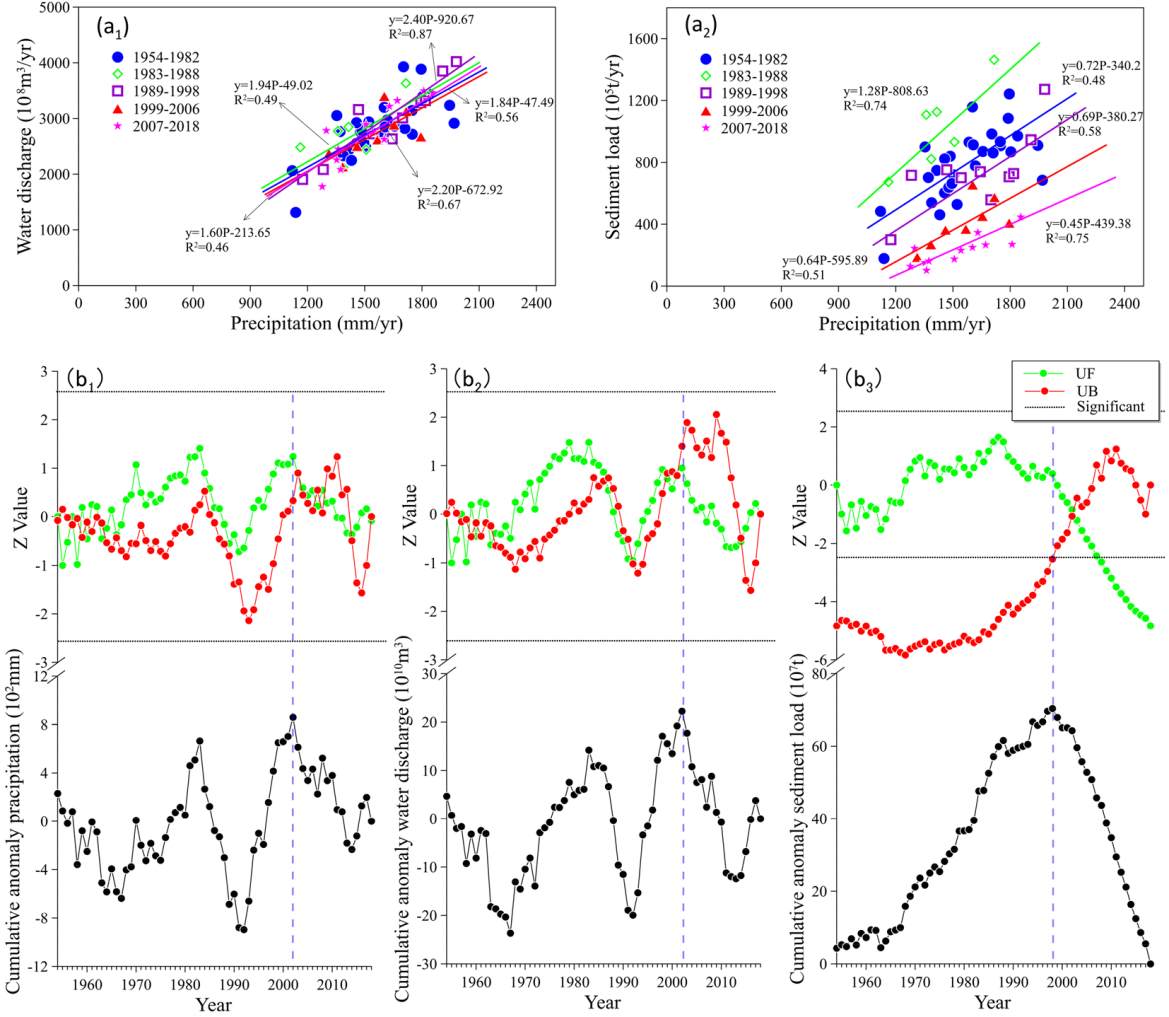
Water discharge and sediment load compared to precipitation during different periods in the Pearl River basin (**a**). Abrupt analysis of precipitation, water discharge and sediment load (**b**).

Climate factors, particularly precipitation, can cause changes in WD and affect the variation in SL. Figure 2 presents the WD trends for the PRB from 1954 to 2018 based on linear regression analysis. The mean annual WD from 1954 to 2018 varied from a minimum value of 1314 × 10^8^ m^3^ in 1963 to a maximum value of 4021 × 10^8^ m^3^ in 1994 with a mean value of 2825 × 10^8^ m^3^. There are no significant increasing trends in the WD time series. According to linear regression analysis, the annual rate of increase in WD is 0.3416 × 10^8^ m^3^/year. When comparing WD to precipitation, it is clear that the fluctuations and tendencies of WD are consistent with variations in precipitation (Fig. 2). The correlation between cumulative annual precipitation and WD exhibits a linear trend for the entire PRB from 1954 to 2018 (Fig. 3b). Figure 4a_1_ also reveals that WD was consistent with precipitation during different decades and maintain synchronization, i.e., precipitation changes result in WD variability in the basin. These results suggest that precipitation is the main explanatory variable for WD in the PR.

Figure 4b_1_,b_2_ present the results of M–K testing and accumulative anomaly curves for annual precipitation and WD, respectively. It is clear that the curves of precipitation and those of WD have similar phases and trends. According to the M–K test results and accumulative anomaly curves, an abrupt change in WD occurred in 2002. Although they are less significant, 1967, 1983, 1992, and 2014 can also be identified as turning points in the WD trend. Note that these turning points largely coincide with global ENSO events. Additionally, the turning points indicate that there were three periods with decreasing trends (1954–1967, 1983–1992 and 2002–2014) and two periods with increasing trends (1968–1982 and 1993–2001). These findings match the perturbations in precipitation over the 6 decades studied. These results suggest that an increase or decrease in WD could be attributed to climate change and natural climatic oscillations (e.g., ENSO and SST).

Regarding the annual SL, although large changing trends were detected from 1954 to 2018, inter-annual fluctuations were consistent with those of precipitation and WD (Fig. 2). Specifically, ENSO years often correspond to low precipitation, resulting in low WD and SL. For example, as shown in Fig. 2, strong ENSO events in 1963 and 2009 coincide with lower precipitation levels of 1138 mm/year and 1374 mm/year, respectively, which correspond to lower WD of 1314 × 10^8^ m^3^/year and 2078 × 10^8^ m^3^/year, and lower SL of 1782 × 10^4^ t/year and 1614 × 10^4^ t/year, respectively. These values are lower than the average decadal precipitation, WD, and SL values in the 1960s and 2000s, respectively. Furthermore, the inter-annual variations in precipitation, WD, and SL at time scales of 2–8 years are consistent with the periodic variations of ENSO^22^. This linkage indicates that changes in ENSO periodicity can affect inter-annual periodic variations in WD and SL. Similar results were also found in Columbia River^30^, Mekong River^31^, Yellow River^32^ and Yangtze River^33^.

The M–K abruptness test and cumulative anomaly test revealed an abrupt change in SL in 1998 (Fig. 4b_3_). The time series for SL can be divided into three phases (a linear phase, increasing phase, and decreasing phase) with turning points in 1982 and 1988 (Fig. 3b_2_). Prior to 1982 (linear phase), the cumulative precipitation and SL are well correlated, and precipitation and SL exhibit a significant relationship. During the periods of 1983–1988 and 1989–2018, SL exhibits a significant increasing and deceasing trends, respectively regardless of precipitation and WD. Figure 4a_2_ also reveals that for the same level of precipitation, the SL increase from 1983 to 1988 and gradually decrease during the periods of 1989–1998, 1999–2006, and 2007–2018. The linear regression equations for annual SL suggest a rate of increase of 1.38 × 10^4^ t/year from 1983 to 1988 and a rate of decrease of 2.24 × 10^4^ t/year from 1989 to 2018. These opposing trends, which independent of precipitation and WD, suggest that there are external controlling mechanisms influencing SL in addition to natural climate change.

### Impact of human activities

The human activities affecting WD and SL include land use change and dam construction. Typically, soil and water loss induced by deforestation results in an increased SL. In contrast, afforestation, soil preservation, and dam construction result in reduced SL. When both positive and negative influences on SL occur in the same period of time, the magnitude of the measurable change in SL depends on the relative balance the two types of factors.

**Figure 5:**
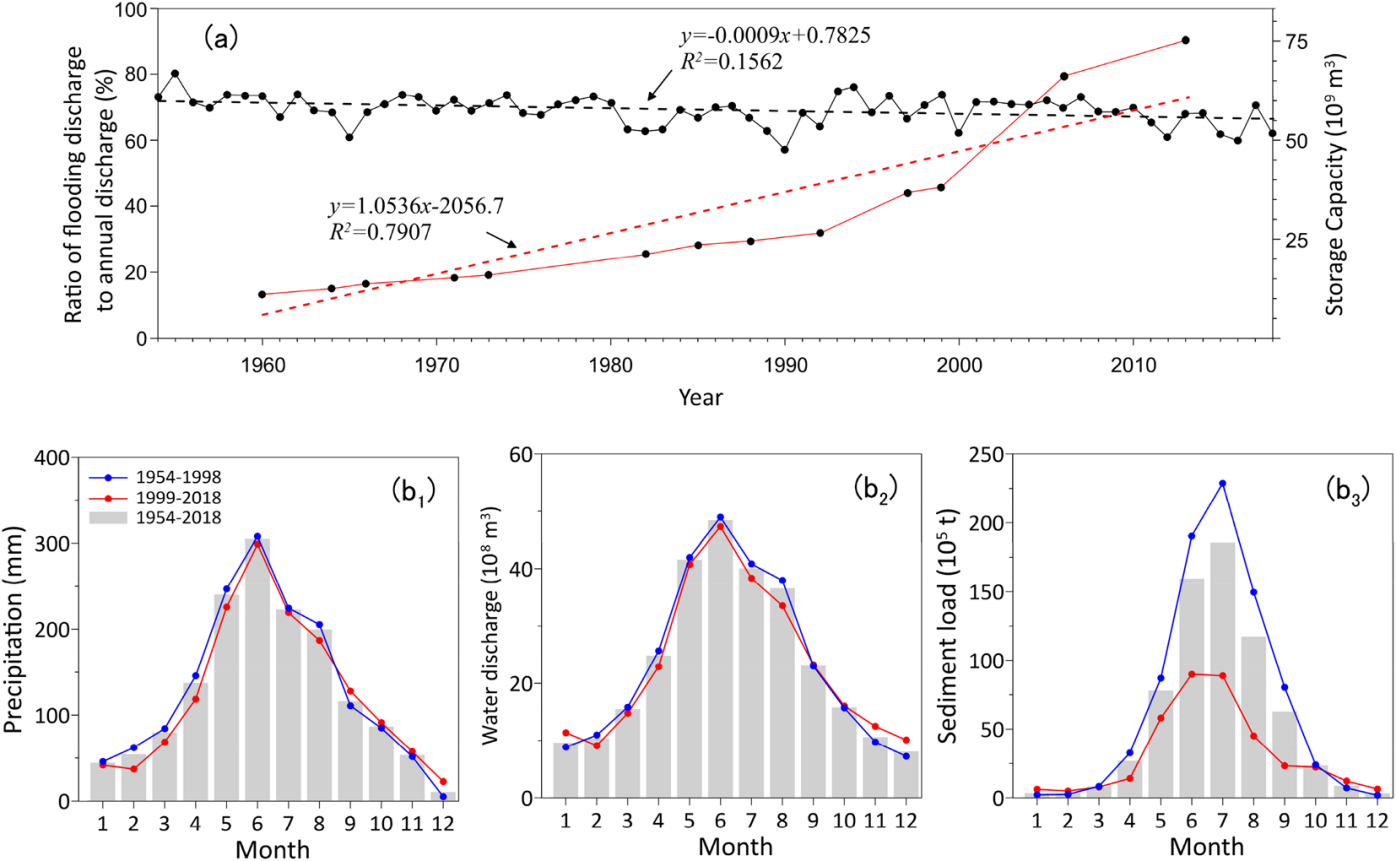
The ratios of flooding discharge to annual water discharge (black line) and total storage capacity (unit in × 10^9^ m^3^) for the reservoirs on the Pearl River (red line) (**a**). Monthly precipitation, water discharge, sediment load, and comparison between pre-abrupt and post-abrupt periods of sediment load in the Pearl River (**b**).

As mentioned previously, the time series of SL in the PR can be divided into three phases. In the first phase (prior to 1982), the SL exhibits no clear changes. This is mainly caused by the balance between deforestation and dam construction. After the end of two major wars (the Second World War and National Liberation War), China entered a period of peace, which resulted in rapid increases in population and deforestation^34^. As a result, soil erosion in the PRB has been speeded up. Although dam construction on the PR began in the 1960s, the decreasing effect of dam construction on SL did not overcome the increasing effect of deforestation in this period. In the second phase from 1983 to 1988, there was a significant increase in SL. The regression relationships indicate that the SL between 1983 and 1988 was approximately 30% higher than that between 1954 and 1982. This trend is mainly caused by the acceleration of soil erosion. At the end of the 1970s, China implemented in a program of agricultural and economic reform. Land was distributed to peasants and crop planting increased significantly, which further accelerated the deforestation. Xia (1999) indicated that the area of soil erosion in the PRB was almost three times higher in the mid-1980s than in the 1950s^35^. In contrast, dam construction in the PRB slowed down in the 1980s^20^. These observations implies that deforestation governed the variation in riverine sediment in this period, which outweigh the influence of dam construction, resulting in an increase in SL. In the third phase from 1989 to 2018, the SL decreased dramatically. The SL in the 2000s and 2010s were only less than half and one third of the 1950s, respectively. This variation, on the one hand, is caused by the efforts to rehabilitate rocky desertified areas, on the other hand, it can be attributed more to an increase in large dam construction. Since the Soil Preservation Law of the Republic of China was enacted in 1991, great efforts have been carried out to prevent soil erosion in Pearl River. According to the remote sensing images of the National Land Use Survey, the area of soil erosion in the PRB in 1995 and 2004 were 62.70 × 10^3^ km^2^ and 62.73 × 10^3^ km^2^, respectively^18^. That is, the area of soil erosion in the PRB was almost unchanged from 1995 to 2004. However, the mean SL in 1995–2004 (54.4 Mt/year) was 30.7% lower than that in 1986–2004 (78.56 Mt/year). Dai et al. (2008) suggested that the area of soil erosion in the PRB was larger in 2000–2005 than in the 1950s and 1960s^18^. However, the SL in 2000–2005 was only half of the 1950s and 1960s. Moreover, the Bulletin of Water and Soil Conservation issued by the Pearl River Water Resource Committee indicated that the total amount of soil erosion in the PRB decreased at a rate of 47.4 Mt/year from 2002 to 2014, which was only a quarter of the rate of deceleration in SL (184.7 Mt/year). These observations implies that although efforts at soil conservation were responsible for the decrease in SL, dam construction was the main factor causing the reduction of SL. Similarly, it has been reported that soil preservation is the second most important factor for decreasing SL in the Yangtze River^33^ and Yellow River^36^ following dam construction. In the PRB, over 9000 dams and reservoirs have been constructed since the 1950s. Figure 3a presents the main large reservoirs with storage capacities exceeding 10^8^ m^3^, all of which are scattered across the PRB. From the 1960s to the 1990s, the total storage capacity of the PRB increased slowly, followed by a boom in dam reservoir constructions after the 1990s (Fig. 5a). Large dams, such as YD, TD, FD and LD, were constructed in the mainstream. Sediment deposition in these dams has been considerable. For example, the deposition rate in the YD reservoir was 35 Mt/year (Dai et al. 2008). The SL recorded at Tian’e hydrologic station decreased by about 30 Mt/year after closure of the TD dam and decreased by a further 30 Mt/year after the closure of the LD dam^20^. From Fig. 3b_1_, one can see that the double mass curve of SL has two notable turning points at approximately 1998 and 2006, which are closely related to the construction of reservoirs in the basin. These observations implies that dam and reservoir constructions have begun to play a dominant role in SL reduction.

Reservoirs typically impound water during the latter half of the wet season (decreasing trend in discharge from August to November) and release water during the driest months (increasing trend in discharge in January and February) to satisfy the demands of agricultural irrigation in the PRB. Based on the seasonal regulation of reservoir water storage in the PRB, the ratios of flood discharge to annual discharge have exhibited a significant decreasing trend over the past 65 years (Fig. 5a). For example, measured WD during flood seasons accounted for more than 78% of the annual WD in the 1950s, but this value decreased to 70% in the 2010s.

Figure 5b presents multiyear averages of monthly precipitation, WD, and SL in different periods based on abrupt changes in SL. Seasonally, the monthly precipitation and WD of the PR are the highest in June and lowest in December and January (Fig. 5b_1_,b_2_). These extreme monthly values occur approximately month earlier than the corresponding values for the Yangtze River^32^. This is likely because the PRB is closer to the ocean (South China Sea) compared to the Yangtze River basin. Therefore, in summer, the southwesterly monsoon winds should transport vapor to the PRB earlier than to the Yangtze River basin. Monthly precipitation and WD exhibit no significant changes between the pre-abrupt and post-abrupt periods in the PRB. However, the SL exhibits a significant difference. The SL in the post-abrupt period is lower than in the pre-abrupt period, with the largest difference occurring in summer (Fig. 5b_3_). This change is a result of dam construction. Furthermore, the SL in winter in the post-abrupt period is greater than that in the pre-abrupt period. This result is largely caused by the seasonal regulation of reservoir water storage.

### Quantitative hydrological responses to climate change and human activities

To quantify the contribution of climate change and human activities on WD and SL variations, a benchmark period must first be defined to represent that this period is mainly dominated by natural processes and the impact of human activities on WD and SL can be negligible. Then, the liner regression method is used to establish the WD and SL for the following periods based on the precipitation data. Finally, the calculated WD and SL can be considered as the contribution of climate change. The difference between measured and the calculated values is considered to be influenced by human activities. As analyzed above, the WD and SL in the PR exhibit no obvious changes prior to 1982. The impact of human activities including dam construction and deforestation during this period was not significant. Therefore, the period from 1954 to 1979 can be used as a benchmark period to quantify the influences of climate change on WD and SL variation. The linear regression equations between the precipitation data and both WD (1) and SL (2), as well as their significance levels, are presented below.

1 $$y = 1.96x - 281 \;\;(R^{2} = 0.96 \;P < 0.01)$$

2 $$y = 8.32x - 5110\;\; (R^{2} = 0.86 \;P < 0.01)$$

**Figure 6:**
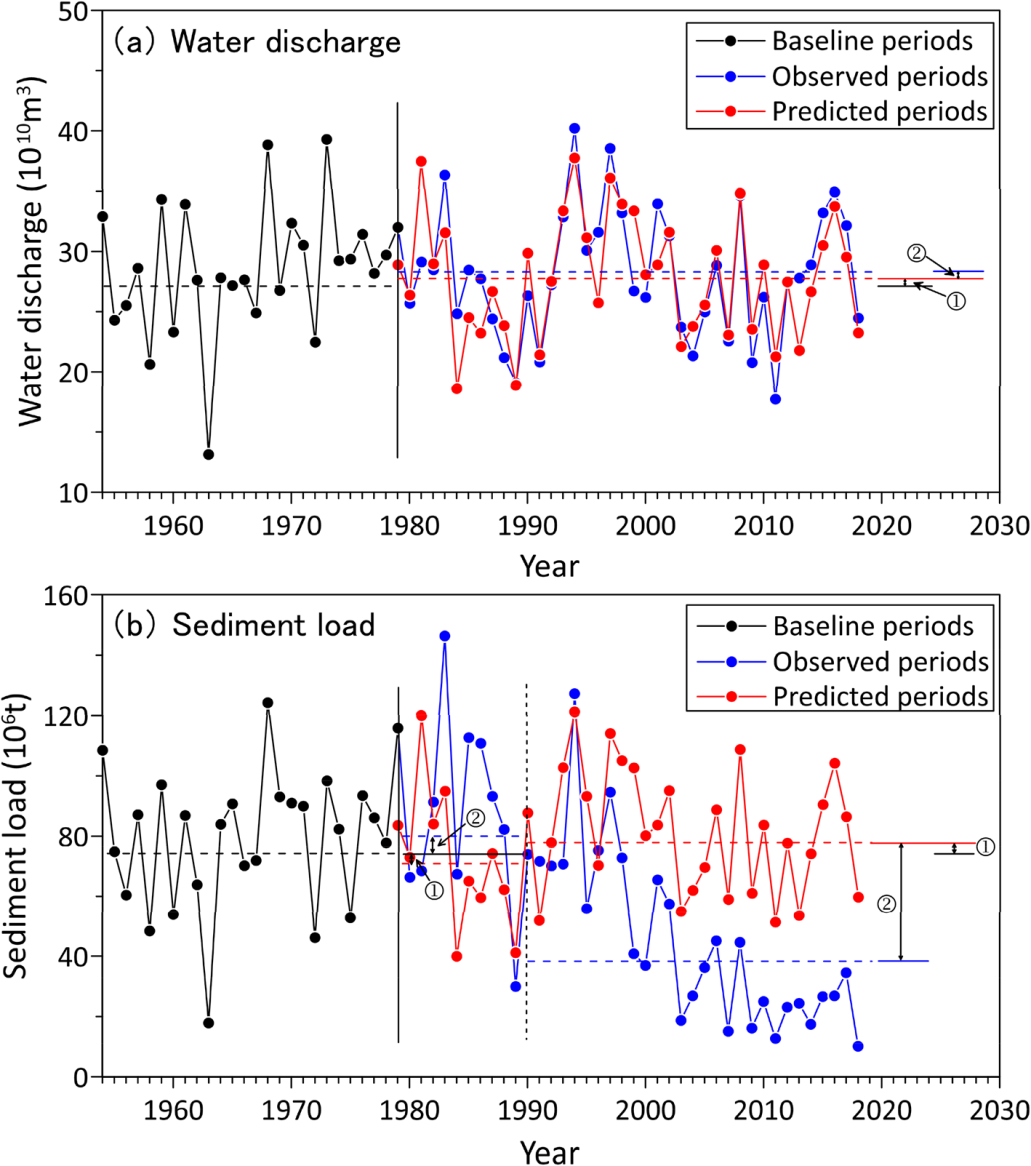
Quantitative estimates of the responses of water discharge (**a**) and sediment load (**b**) to climate change and human activities in the Pearl River basin. Intervals and indicate the impacts of climate change and human activities, respectively.

Figure 6 presents the baseline data, reconstructed data, and observed data for WD and SL in the PRB. Interval 1 represents the impact of climate change (mainly precipitation in the PRB); while Interval 2 represents the impact of human activity (primarily dam construction, land-use change, water abstraction and irrigation). Regarding WD (Fig. 6a), the predicted annual values are in agreement with the observed data in terms of extreme values throughout the period of observation, which indicates that the WD in the PR is ultimately controlled by climate change. Regarding SL (Fig. 6b), the predicted values are generally lower than the measured values in the 1980s, while since the late 1990s, the measured values began to be significantly lower than the predicted values. Both of these changes are mainly controlled by human activities. As analyzed above, large-scale deforestation in the 1980s aggravated erosion in the watershed and contributed to the increase in SL, while the implementation of soil conservation and the massive construction of large dams after the 1990s resulted in a sharp decrease in the SL delivered to the ocean. Table 1 lists the decadal variations in mean annual precipitation, mean annual WD, and mean annual SL, as well the percentage differences with respect to the baseline values. At baseline, the reconstructed WD (2790.3 × 10^8^ m^3^/year) and SL (7955.01 × 10^4^ t/year) were close to the observed data (2763.6310^8^ m^3^/year in WD and 7783.88 × 10^4^ t/year in SL), with errors of less than 1% and 2.2%, respectively, which also supported the simulated values obtained for the 1980s to 2010s. As shown in Table 1, Precipitation was positively correlated with reconstructed WD and SL. During the 1980s, 2000s and 2010s, decadal precipitation decreased in drainage areas, which had a positive effect on the generation of runoff, which directly increased the reconstructed WD and SL data. During 1990s, precipitation increased by 8% relative to the baseline, resulting in increases in reconstructed WD and SL by 11% and 17%, respectively. However, the measured SL was negatively correlated with rainfall during 1980s and 1990s. In addition, the reduction in measured SL were far greater than that of precipitation during 2000s and 2010s. For example, in the 2010s, the precipitation decreased by 3% relative to the baseline while the measured SL decreased by 72%.

**Table 1:** Decadal variations of precipitation, water discharge and sediment load in the Pearl River basin*.

Period	Precipitation (mm/year)	Water discharge (× 10^8^ m^3^/year)		Sediment load (× 10^4^ t/year)	
		Observation	Prediction	Observation	Prediction
Baseline	1561.39	2763.63	2790.3	7783.88	7955.01
1980s	1494.08 (− 4%)	2659.91 (− 4%)	2601.62 (− 7%)	8763.52 (+ 13%)	7139.77 (− 10%)
1990s	1685.63 (+ 8%)	3075.01 (+ 11%)	3101.96 (+ 11%)	7156.08 (− 8%)	9623.25 (+ 17%)
2000s	1531.70 (− 2%)	2683.19 (− 3%)	2716.15 (− 3%)	3504.31 (− 55%)	7625.84 (− 4%)
2010s	1511.96 (− 3%)	2683.98 (− 3%)	2701.62 (− 3%)	2195.88 (− 72%)	7564.17 (− 5%)

*Data in parentheses indicate relative changes compared to the corresponding values in the baseline periods; the symbol “+” corresponds to a means increase, whereas “−” corresponds to a mean decrease.

Table 2 summarizes the contributions of climate change and human activities to changes in WD and SL during different periods. Regarding WD, the greatest change occurred during the 1990s, where the value increased by 311.38 × 10^8^ m^3^/year relative to the baseline. Climate change and human activities contributed + 109% and − 9% of this increase, respectively. The most significant impacts of human activity on WD occurred in the 1980s, where the contribution of human activities to WD increase was 56%. Deforestation in the watershed will lead to soil erosion and water loss, which will increase water discharge. Generally, in the PRB, climate change contributes much more to hydrological factors, regardless of whether its effects are negative or positive. For example, during 2010s, climate change and human activities contributed 78% and 22% to the reduction in WD, respectively. Regarding SL, the greatest increase occurred in the 1980s, where the value increased by 979.64 × 10^4^ t/year relative to the baseline. The contribution of human activities to this increase was 166%. SL decreased from the 1990s to 2010s. The SL decrease caused by human activities was 2107.17 × 10^4^ t/year in the 1990s, 4121.53 × 10^4^ t/year in the 2000s, and 5638.39 × 10^4^ t/year in the 2010s. The contribution of human activities varies from 96 to 336%, with the most obvious increase occurring in the 1990s. During 2010s, the contribution of climate change and human activities to decrease in SL were 4% and 96%, respectively. These results suggest that the impact of human activities on SL exceeds that of climate change, meaning human activities play a dominant role in terms of sediment change.

**Table 2:** Quantification of effects*.

Period	Water discharge (× 10^8^ m^3^/year)			Sediment load (× 10^4^ t/year)		
	Total change	Climate change	Human activities	Total change	Climate change	Human activities
1980s	− 103.72	− 162.01 (+ 156%)	58.29 (− 56%)	979.64	− 644.11 (− 66%)	1623.75 (+ 166%)
1990s	311.38	338.33 (+ 109%)	− 26.95 (− 9%)	− 627.80	1479.37 (− 236%)	− 2107.17 (+ 336%)
2000s	− 80.45	− 47.48 (+ 59%)	− 32.96 (+ 41%)	− 4279.57	− 158.04 (+ 4%)	− 4121.53 (+ 96%)
2010s	− 79.65	− 62.01 (+ 78%)	− 17.64 (+ 22%)	− 5588.00	− 219.71 (+ 4%)	− 5638.29 (+ 96%)

*Data in parentheses indicate relative changes with respect to the total change; the symbol “+” represents a positive effect on total change, whereas “−” represents a negative effect on total change.

## Discussion

The spatiotemporal variation of precipitation will have an important impact on the spatiotemporal distribution of water resources, leading to severe floods or droughts. In addition to the strong ENSO event, which has significant impacts on regional precipitation in the PRB, other global climate changes and large-scale ocean–atmosphere processes, such as Pacific Decadal oscillation (PDO), Indian Ocean dipole (IOD), and North Atlantic oscillation (NAO), also influence the hydrological cycle of the PR^37,38^. In particular, these large-scale ocean–atmosphere processes will interact and adjust to varying degrees, making the precipitation processes in the PRB more highly complex. Therefore, more attention needs to be paid to the correlation between these processes and their impact on precipitation and WD in future studies.

Among the human activities in the watershed, dam construction is the most direct way to manipulate the SL. However, the impact of the dam on SL depends not only on its storage capacity, but also on its location on the river, which determines the catchment area, water discharge, and whether there are other dams. In the PRB, although many dams were constructed in the 1950s–1980s, their impact on the SL is not significant because most of them have small storage capacity and are located on tributaries. A few dams with large storage capacity also have relatively limited impact on SL due to the small catchment area. For example, the XFD dam (Fig. 3a), built in 1960, is the oldest and, until now, the second largest dam in the PRB, with a reservoir storage capacity of 138.9 × 10^9^ m^3^. However, it was constructed across a tributary of the East River (Xinfeng River), where the catchment area is just 5.7 × 10^3^ km^2^ and the SL before its operation was only 0.8 Mt/year. Therefore, the impact of the dam on SL in the PR is not significant. Since the beginning of the 1990s, dam construction in the PRB has accelerated significantly. As shown in Fig. 5a that the total storage capacity of reservoirs increased sharply and nearly tripled in the 2000s when compared to the capacity in the 1980s. Many large dams, such as YD, TD, FD and LD, were constructed on the mainstream of the rivers (Fig. 3a). Since then, sediment deposition in these more recent reservoirs had begun to play a dominant role, and resulted in a decrease in the SL delivered to the delta. Therefore, future dam construction in the PRB needs to take this into account.

Riverine sediment discharge into the sea has been a topic of global concern in recent years. Knowledge regarding sediment flux is of great significance not only for determining the accuracy of multi-year river sediment data^39^, but also for understanding the evolution of deltas and estuaries, as well as coastal environments^34^. Dramatic decreases in sediment discharge accelerate delta and shoreline recession, which has been observed in many deltas, such as the Nile River delta^40^, Ebro River delta^41^, Red River delta^42^, Mekong River delta^31^, Yellow River delta^43^, and Yangtze River delta^44^. Therefore, it is reasonable to conclude that anthropogenic impacts on WD and SL have changed the evolutional pattern of the Pearl River delta and its coast. Recently, a deceleration of the delta growth rate has been reported in the Pearl River delta, although the general trend is still prograding^45^. Most river outlets began eroding in the 1990s to 2000s, rather than silting up as they had previously^14^. Although there are other factors contributing to these changes, such as in-channel sediment mining and unreasonable coastal construction, the decline of SL is a dominant factor. Furthermore, given the large number of reservoirs in the PRB and their enormous storage capacity, the SL in the main river is expected to remain low on a century timescale^16,18^. Therefore, in future scientific research and management projects, increased attention must be paid to the long-term effects of a reduction in sediment flux on environmental changes. Furthermore, the remarkable variation in SL in the PR combined with nearly unchanged levels of WD (Fig. 2) is also a good example of the effects that human activities can have on a river system.

## Conclusions

The SL of the PR exhibited an increasing trend in 1980s and has exhibited a significant decreasing trend since 1990s, while the WD of the river did not show any significant trend. The observation implies that the impact of deforestation on increasing SL in the 1980s has been more than compensated by dam construction which have reduced sediment conveyance. Since 1990s, thanks to the efforts of the water and soil conservation, and more importantly, to the boom of constructions of large dams, the SL into the sea declines dramatically. Quantitative analysis revealed that human activities and climate change are responsible for 96% and 4% of the decreases in SL, respectively. With more dam construction and an intensification of the afforestation policy in the drainage basin, the SL of the PR is expected to decrease further. Decrease in sediment supply from river may have a serious impact on the ecosystem and evolution of the delta, which requires further studies in the future.

## Study area

The PRB covers the area ranging from 21.31° to 26.49° N and 102.14° to 115.53° E with a drainage area of 0.45 × 10^6^ km^2^ (Fig. 1). It covers a region of subtropical to tropical monsoon climates straddling the Tropic of Cancer. The annual mean temperature across the basin is 14–22 °C, and the mean annual precipitation ranges from 1200 to 2200 mm^18^. The PR is a compound river system, and comprises three major tributaries: West River, North River, and East River, as well as other small rivers that drain into the Pearl River estuary (Fig. 1). The West River is the largest tributary with a length of 2214 km, a basin area of 0.35 × 10^6^ km^2^, accounting for 77.8% of the total drainage area of the Pearl River Basin. The North River is the second largest tributary, with a length of 468 km and a drainage area of 0.46 × 10^5^ km^2^. With a basin area of 0.27 × 10^5^ km^2^, the East River (length = 562 km) has the lowest water discharge and sediment load of the three tributaries. The ‘Pearl River’ water and sediment data in this study are defined as the sum of these three tributaries. Influenced by the subtropical monsoon climate, water discharge levels from the Pearl River fluctuate seasonally and significantly. Water discharge and sediment load during the flood season (April–September) account for ~ 78% and ~ 98% of the total annual amount, respectively.

## Methods

The linear regression method was used to analyze the trends precipitation, water discharge and sediment load from 1954 to 2018. The Mann–Kendall test method^13^ and cumulative anomaly method^38^ were applied to detect any abrupt changes in annual precipitation, water discharge and sediment load. The double mass curve method^23^ was used to estimate the relative effects of climate change and human activities on water discharge and sediment load in the basin.

## References

[1] Walling DE, Fang D (2003). Recent trends in the suspended sediment loads of the world's rivers. Glob. Planet. Change.

[2] Wang HJ (2011). Recent changes of sediment flux to the western Pacific Ocean from major rivers in east and Southeast Asia. Earth Sci. Rev..

[3] Sun P (2020). Shifts of sediment transport regime caused by ecological restoration in the Middle Yellow River Basin. Sci. Total Environ..

[4] Syvitski JP, Vörösmarty CJ, Kettner AJ, Green P (2005). Impact of humans on the flux of terrestrial sediment to the global coastal ocean. Science.

[5] Walling DE (2006). Human impact on land-ocean sediment transfer by the world's rivers. Geomorphology.

[6] Milliman JD (2008). Climatic and anthropogenic factors affecting river discharge to the Global Ocean, 1951–2000. Glob. Planet. Change.

[7] Hansen J (2006). Global temperature change. P. Natl. Acad. Sci. U.S.A..

[8] Piao SL (2007). Changes in climate and land use have a larger direct impact than rising CO_2_ on global river runoff trends. Proc. Natl. Acad. Sci. U.S.A..

[9] Labat D, Goddéris Y, Probst JL, Guyot JL (2004). Evidence for global runoff increase related to climate warming. Adv. Water Resour..

[10] Blum MD, Roberts HH (2009). Drowning of the Mississippi Delta due to insufficient sediment supply and global sea-level rise. Nat. Geosci..

[11] Li X (2017). Recent evolution of the Mekong Delta and the impacts of dams. Earth Sci. Rev..

[12] Kong D (2015). Evolution of the Yellow River Delta and its relationship with runoff and sediment load from 1983 to 2011. J. Hydrol..

[13] Du JL, Yang SL, Feng H (2016). Recent human impacts on the morphological evolution of the Yangtze River Delta foreland: a review and new perspectives. Estuar. Coast. Shelf Sci..

[14] Zhang W (2015). Morphological change in the Pearl River Delta, China. Mar. Geol..

[15] Qiu L (2017). Spatiotemporal response of the water cycle to land use conversions in a typical hilly–gully basin on the Loess Plateau, China. Hydrol. Earth Syst. Sci..

[16] Wu C (2019). The impact of climate change and human activities on streamflow and sediment load in the Pearl River basin. Int. J. Sediment Res..

[17] Sun P (2020). Quantifying the contributions of climate variation, land use change, and engineering measures for dramatic reduction in streamflow and sediment in a typical loess watershed, China. Ecol. Eng..

[18] Dai SB, Yang SL, Cai AM (2008). Impact of dams on the sediment flux of the Pearl River, Southern China. CATENA.

[19] Zhang SR (2008). Recent changes of water discharge and sediment load in the Zhujiang (Pearl River) Basin, China. Glob. Planet Change.

[20] Wu CS, Yang SL, Lei YP (2012). Quantifying the anthropogenic and climatic impacts on water discharge and sediment load in the Pearl River (Zhujiang), China (1954–2009). J. Hydrol..

[21] Zhang Q, Xu CY, Chen XH, Lu XX (2012). Abrupt changes in the discharge and sediment load of the Pearl River, China. Hydrol. Process..

[22] Liu F (2017). Impacts of ENSO on multi-scale variations in sediment discharge from the Pearl River to the South China Sea. Geomorphology.

[23] Dettinger MD, Cayan DR, McCabe GJ, Marengo JA, Diaz HF, Markgraf V (1999). Multiscale hydrologic variability associated with El Niño -Southern Oscillation. El Niño and the Southern Oscillation: Multiscale Variability and Global and Regional Impacts.

[24] Ward PJ (2010). Sensitivity of river discharge to ENSO. Geophys. Res. Lett..

[25] Wanders N, Wada Y (2015). Human and climate impacts on the 21st century hydrological drought. J. Hydrol..

[26] Lee T, McPhaden MJ (2010). Increasing intensity of El Niño in the central-equatorial Pacific. Geophys. Res. Lett..

[27] Glantz MH, Katz RW, Nicholls N (1991). Teleconnections Linking World Wide Climatic Anomalies.

[28] Niu J (2013). Precipitation in the Pearl River basin, South China: scaling, regional patterns, and influence of large-scale climate anomalies. Stoch. Environ. Res. Risk Assess..

[29] Zhao Y, Zou X, Cao L, Xu X (2014). Changes in precipitation extremes over the Pearl River Basin, southern China, during 1960–2012. Quatern. Int..

[30] Naik PK, Jay DA (2011). Distinguishing human and climate influences on the Columbia River: changes in mean flow and sediment transport. J. Hydrol..

[31] Xue Z, Liu JP, Ge Q (2011). Changes in hydrology and sediment delivery of the Mekong River in the last 50 years: connection to damming, monsoon, and ENSO. Earth Surf. Process. Landf..

[32] Wang HJ (2006). Inter-annual and seasonal variation of the Huanghe (Yellow River) water discharge over the past 50 years: connections to impacts from ENSO events and dams. Glob. Planet. Change.

[33] Zhao YF (2015). Quantifying the anthropogenic and climatic contributions to changes in streamflow and sediment load into sea: a case study of the Yangtze River, China. Sci. Total Environ..

[34] Yang SL, Zhao QY, Belkin IM (2002). Temporal variation in the sediment load of the Yangtze River and the influences of the human activities. J. Hydrol..

[35] Xia HP (1999). Flood disasters, soil erosion, and eco-restoration of vegetation in the Yangtze and the Pearl River Valleys. Trop. Geogr..

[36] Miao C, Ni J, Borthwick AGL, Yang L (2011). A preliminary estimate of human and natural contributions to the changes in water discharge and sediment load in the Yellow River. Glob. Planet. Change.

[37] Wang L, Chen W, Huang RH (2008). Interdecadal modulation of PDO on the impact of ENSO on the East Asian winter monsoon. Geophys. Res. Lett..

[38] Chan JCL, Zhou W (2005). PDO, ENSO and early summer monsoon rainfall over south China. Geophys. Res. Lett..

[39] Sun P (2019). Can the Grain-for-Green Program really ensure a low sediment load on the Chinese Loess plateau?. Engineering.

[40] Fanos AM (1995). The impact of human activities on the erosion and accretion of the Nile delta coast. J. Coast. Res..

[41] Mikhailova MV (2003). Transformation of the Ebro River Delta under the impact of intense human-induced reduction of sediment and runoff. Water Resour..

[42] Dang TH (2010). Long-term monitoring (1960–2008) of the river-sediment transport in the Red River Watershed (Vietnam): temporal variability and dam-reservoir impact. Sci. Total Environ..

[43] Yu J (2011). Effects of water discharge and sediment load on evolution of modern Yellow River Delta, China, over the period from 1976 to 2009. Biogeosciences.

[44] Yang ZS (2006). Dam impacts on the Changjiang (Yangtze) River sediment discharge to the sea: the past 55 years and after the Three Gorges Dam. Water Resour. Res..

[45] Wu Z (2018). Geomorphologic changes in the lower Pearl River Delta, 1850–2015, largely due to human activity. Geomorphology.

